# Sexual functioning in trichotillomania and skin picking disorder

**DOI:** 10.3389/fpsyt.2026.1799416

**Published:** 2026-06-17

**Authors:** Megha Neelapu, Jon E. Grant

**Affiliations:** Department of Psychiatry & Behavioral Neuroscience, University of Chicago, Pritzker School of Medicine, Chicago, IL, United States

**Keywords:** body-focused repetitive behaviors, sexual dysfunction, sexual functioning, skin picking disorder, trichotillomania

## Abstract

**Background:**

This study examines sexual functioning in adults with trichotillomania and skin picking disorder. To our knowledge, no study has examined sexual functioning in either of these disorders.

**Methods:**

334 adults (mean age=30.21, SD = 8.23, 83.5% cisgender women, 67.6% sexually active with another person in the past month) were recruited from online Reddit communities. Participants completed surveys assessing demographics, sexual functioning, and current and lifetime pulling and picking severity. Comorbidities and treatment history were assessed via dichotomous variables. Sexual functioning was assessed via the Changes in Sexual Functioning Questionnaire – Short Form (CSFQ-14).

**Results:**

27.1% (n=85) of the sample met criteria for sexual dysfunction. After controlling for relationship status, participants who rated their picking as currently at its worst had significantly lower scores on the Orgasm/Completion subscale. Sexual dysfunction, the total score, and subscales scores were not significantly associated with any current comorbidities or treatment variables.

**Conclusions:**

It is difficult to compare rate of sexual dysfunction in adults with trichotillomania and skin picking disorder with the rate in the general population given the homogeneity of this study’s sample. However, worse skin picking symptoms appear to be associated with worse sexual functioning, particularly in the orgasm domain. Since severity of comorbidities was not assessed in this study, further research is needed to determine whether comorbid disorders have an effect on sexual functioning in this population.

## 1 Introduction

This study aims to examine sexual functioning in adults with trichotillomania and skin picking disorder. To our knowledge, no study has examined sexual functioning in either of these disorders.

The most frequent sexual problems in women tend to be low sexual interest, issues with arousal and lubrication, and orgasmic dysfunction. In men, they are premature ejaculation and orgasmic dysfunction (1). Common cross-cultural predictors of sexual dysfunction include relationship dissatisfaction, older age, lower educational attainment, negative experiences in sexual relationships, reduced quality of life, and physical and mental health problems (2–6).

Systematic reviews and meta-analyses show that people with psychiatric disorders generally have a greater rate of sexual dysfunction, although the prevalence and types of sexual problems vary depending on the disorder (7–9). Risk factors found to be associated with sexual dysfunction in psychiatric populations include longer duration of illness, history of relapse, and use of antipsychotic medications (7). A meta-analysis of studies on sexual functioning and depression also demonstrated that there is a bidirectional relationship between depression and sexual dysfunction, which suggests that psychiatric illness may be both a cause and an effect of sexual dysfunction (10).

The specific mechanics by which psychiatric disorders affect sexual dysfunction and vice versa is unclear. For example, in OCD—a closely related disorder to trichotillomania and skin picking disorder—it does not appear that the severity or type of OCD is correlated with sexual dysfunction (11, 12). Some have attributed increased sexual dysfunction in those with OCD to lower frequency of sexual experiences with others (13, 14). Moreover, because better mental health predicts entry into romantic relationships, those with psychiatric disorders may overall be less likely to be in relationships and have sexual experiences with others (15). However, Vulink et al. (12) still reported higher rates of sexual problems in women with OCD compared to a healthy control group with similar levels of sexual experiences with others, including lower sexual desire, more sexual disgust, less sexual arousal, and less satisfying orgasms.

Researchers have also suggested that psychiatric medications may be a cause of sexual dysfunction in psychiatric populations, considering that many patients begin reporting sexual problems after starting medication (13). However, existing studies show that the prevalence of sexual dysfunction is elevated even in those with psychiatric disorders who are not taking any medications (8, 9, 12, 16). Further research is still needed here given that these results could be explained by medications preventing improvements in sexual functioning in treated patients (16).

There may also be third factors contributing to sexual dysfunction in those with psychiatric disorders. Psychiatric populations have more relationship problems, and relationship satisfaction is most likely the strongest predictor of sexual functioning (13, 15, 17). Relationship satisfaction, psychiatric symptoms, and sexual dysfunction may all have both direct and indirect effects on each other. It could also be that there are certain traits associated with psychiatric disorders that affect sexual performance itself or lead to sexual dysfunction because of their effect on relationships. For example, in those with OCD, a need for certainty, perfectionism, and avoidance of potentially distressing experiences may lead to relationship problems and subsequently sexual dysfunction (13, 14, 18). There is some evidence that those with OCD have a higher rate of sexual dysfunction even before the onset of OCD symptoms (19). Therefore, one potential explanation of this phenomenon is that there are related traits that are present before the onset of psychiatric symptoms which affect sexual functioning. Another possible explanation is that the developmental course of OCD affects sexual development, given that one study found that men with OCD have a lower age at first masturbation and first nocturnal emission (13). These differences in sexual development may then affect sexual functioning, although more research is necessary in this area.

Trichotillomania and skin picking disorder may be linked to sexual dysfunction in multiple ways. Similar to other psychiatric illnesses, distress and functional interference from these disorders could both directly lead to dysfunction and indirectly lead to dysfunction by contributing to relationship problems. Use of psychiatric medications in this population, whether for their pulling or picking behaviors or for comorbid disorders, may also increase sexual dysfunction. Finally, there are factors specific to trichotillomania and skin picking disorder that could impact sexual functioning. Both hair pulling and skin picking often lead to unwanted changes in one’s physical appearance, including visible hair loss, skin damage, and scarring. People with these disorders can feel more self-conscious about their physical appearance (20–22). Concerns about appearance may in turn lead to heightened anxiety during sexual experiences with others and thus sexual dysfunction (23, 24). Moreover, the developmental course of these disorders could be tied to sexual functioning. The age of onset for trichotillomania tends to be around puberty, which is typically also the age of onset for major aspects of sexual development (25, 26). Distress from hair pulling behaviors may impact sexual functioning at this key developmental point, which could then lead to sexual dysfunction into adulthood. It has also previously been hypothesized that lower levels of sex hormones in adolescence is a contributing factor in the development of trichotillomania, which could simultaneously affect sexual functioning (27).

Overall, the existing literature shows that psychiatric disorders are correlated with sexual dysfunction, but the nature of the relationship is complex and requires further research. Moreover, prior studies on psychiatric illness and sexual dysfunction tend to focus on depressive disorders, anxiety disorders, OCD, and schizophrenia, but there have been few studies on other disorders (9). While there are several ways that trichotillomania and skin picking disorder may be tied to sexual functioning, there is little published research to support these claims. The current study intends to add to the literature by examining sexual functioning in adults with trichotillomania and skin picking disorder and variables associated with sexual functioning in this population.

## 2 Methods

### 2.1 Participants

Adults between the ages of 18 to 65 years were recruited from online Reddit communities for trichotillomania and skin picking disorder as well as other body-focused repetitive behaviors (e.g., nail biting). Participants completed surveys assessing demographics, sexual functioning, current and lifetime pulling and picking severity, current comorbidities, and current treatments.

Participants self-identified as having trichotillomania and/or skin picking disorder. To ensure that participants engaged in an adequate level of hair pulling or skin picking behavior, participants were only included if they endorsed pulling or picking 3 or more days in the past week, more than 15 minutes per day, and said that their pulling or picking either bothered them or interfered with their daily life over the past week (distress or interference rated at least 1 on a scale of 0 to 5). Furthermore, participants who selected “asexual” as their sexual orientation (n=12) were excluded from analyses, given that the CSFQ-14 is not intended for individuals for whom little to no sexual desire is reflective of their normal sexual functioning rather than dysfunction. This brought the final sample size to 334.

All study procedures received approval from the University of Chicago’s Institutional Review Board (IRB) and were done in compliance with ethical guidelines outlined in the Declaration of Helsinki. Participants were required to read and sign an online informed consent before they were allowed to move forward in the survey. Survey completers could enter a raffle to win a $100 virtual Visa gift card. 15 winners were randomly selected.

### 2.2 Measures

Participants self-reported demographic variables as well as current sexual activity. Current sexual activity was defined as having sexual experiences with at least one other person in the past month. Sexual experiences were defined as any kind of sexual acts with other people, including kissing, touching, intercourse, or other sexual acts.

Sexual functioning was assessed via the Changes in Sexual Functioning Questionnaire – Short Form (CSFQ-14) (28). The scale consists of 14 items that ask about sexual functioning over the past month across five domains: Desire/Frequency, Desire/Interest, Pleasure, Arousal/Excitement, and Orgasm/Completion. Participants answer questions on a Likert scale from 1 to 5. Total score ranges from 14 to 70, with higher scores representing better sexual functioning. Scores below or equal to 47 in males and below or equal to 41 in females indicate sexual dysfunction. The scale also results in five subscale scores based on its five domains.

Current pulling and picking severity was measured over the past week by number of days per week spent pulling or picking, time spent per day pulling or picking (in minutes), how much their pulling or picking bothered them on a scale of 0 to 5, and how much their pulling or picking interfered with their daily life on a scale of 0 to 5 (0=not at all, 5=very, very much). For days spent pulling or picking in the past week and time spent per day puling or picking, participants wrote in their answers. These severity items were in part based off of the National Institute of Mental Health Trichotillomania Symptom Severity Scale (NIMH-TSS), which similarly asks participants how much time they spent pulling hairs in a typical day (in minutes), how much their hair pulling bothers them on a scale of 0 to 5, and how much their hair pulling interferes with their daily life on a scale of 0 to 5 (29). To measure lifetime pulling and picking severity, participants were asked to report on the same variables when their pulling or picking was the worst in their lifetime.

To evaluate comorbidities, participants were asked “Have you ever been diagnosed with any of the following disorders (check all that apply)?” The options were, “I have never had a psychiatric disorder,” “depression,” “any anxiety disorder (e.g., generalized anxiety, social anxiety, panic disorder, phobias),” “PTSD,” “ADHD,” “OCD,” “bipolar disorder,” “schizophrenia,” “any personality disorder (e.g., borderline, narcissistic, avoidant),” “eating disorder,” “alcohol use disorder,” “substance use disorder,” and “other.” Participants were then asked if they were currently experiencing symptoms of each disorder that was checked. To assess current treatments, participants were asked to indicate if they were currently taking psychiatric medications, hormonal medications, and if they were in psychotherapy. If they indicated that they were currently taking psychiatric medications, they were asked to write out what medications they were taking. This data was then coded into medication classes (e.g., antidepressants, stimulants).

### 2.3 Statistical analyses

This study aimed to identify variables associated with sexual dysfunction based on the CSFQ-14 cutoff scores. It also aimed to identify variables associated with the degree of overall sexual functioning according to the CSFQ-14 total score and the degree of sexual functioning in each domain of sexual functioning according to the subscale scores. T-tests, ANOVAs, chi-square tests, and Pearson correlations were conducted for these statistical analyses. When it was necessary to control for demographic variables, ANCOVAs and partial correlations were performed as applicable. Effect sizes were categorized using cutoffs into either small (d=0.20, φ=0.10, η_p_^2^ = 0.01), medium (d=0.50, φ=0.30, η_p_^2^ = 0.06), or large (d=0.80, φ=0.50, η_p_^2^ = 0.14).

There is some missing data in this study, given that participants were allowed to skip items they did not feel comfortable answering. This includes the CSFQ-14, where 20 participants did not answer at least 1 item. Therefore, these participants’ data is analyzable for some subscales but not others, and it was not analyzable for the total score. Given our large sample size, we do not believe missing data introduced bias into the results.

IBM SPSS Version 31.0 was used for all data analysis. Considering that this study is exploratory, it was determined that a Bonferroni correction would be overly restrictive to control for multiple comparisons. Instead, the significance level was set at p-values <0.01 to be considered statistically significant.

## 3 Results

### 3.1 Demographics

The final sample consisted of 334 participants ages 18 to 65 (mean age=30.21, SD = 8.23). 27.2% of the sample had trichotillomania, 56.0% had skin picking disorder, and 16.8% had both. 83.5% were cisgender women, 9.0% were cisgender men, and 7.5% were gender minorities who were assigned female at birth. 71.8% were non-Hispanic White, 59.0% were heterosexual, 59.0% worked full-time, and 68.5% had a bachelor’s degree or higher. 64.7% were in a relationship, 10.2% were dating or “hooking up” (having sexual experiences with others outside of the bounds of dating or a relationship), and 25.1% were single (not dating or having sexual experiences with other people). In the past month, 63.4% had sexual experiences with one person, 4.2% with multiple people, and 32.4% with no one. Therefore, 67.6% of the sample were considered currently sexually active.

**Figure 1:**
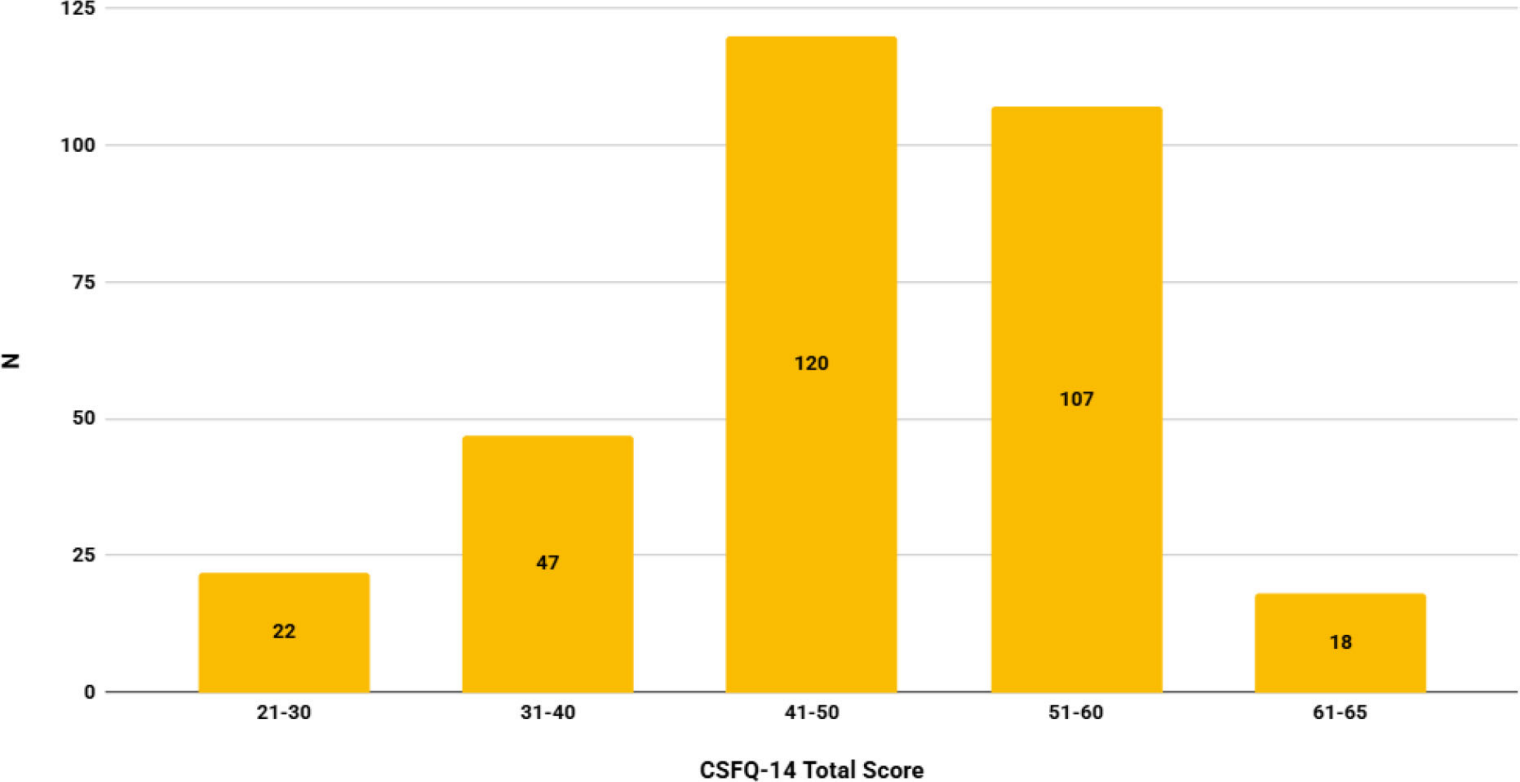
CSFQ-14 total scores for 314 participants with trichotillomania, skin picking disorder, or both.

Based on the CSFQ-14, 27.1% of the full sample met criteria for sexual dysfunction. There was no significant difference in rates of sexual dysfunction between those who had trichotillomania (22.5%), skin picking disorder (25.9%), and both (38.2%). There were also no significant differences in the total or subscale scores by disorder. The mean total score was 46.30 (SD = 9.27), 95% CI [45.27, 47.33] (**Figure 1**). There was no significant difference in age between those who met criteria for sexual dysfunction (M = 31.72, SD = 10.75) than those who did not (M = 29.61, SD = 7.08). Age was also not associated with the CSFQ-14 total or subscale scores.

There was no significant differences in rates of sexual dysfunction by sex and gender (cisgender women: 28.6%, gender minorities: 25.0%, cisgender men: 14.3%). Cisgender women had significantly lower total scores (M = 45.65, SD = 9.48) than cisgender men (M = 53.32, SD = 5.82), t(44.069)=-6.157, p<.001, d=-.835, (large effect size). Gender minorities also had significantly lower total scores (M = 45.25, SD = 6.65) than cisgender men (M = 53.32, SD = 5.82), t(50)=4.667, p<.001, d=1.298, (large effect size). In terms of the specific domains on the CSFQ-14, cisgender women had significantly lower scores on the Desire/Frequency [t(306)=-3.163, p=.002, d=-.608], Desire/Interest [t(305)=-4.792, p<.001, d=-.921], and Arousal/Excitement [t(299)=-6.448, p<.001, d=-1.260] subscales than cisgender men. Gender minorities also had significantly lower scores on the Arousal/Excitement subscale than cisgender men, t(51)=5.139, p<.001, d=1.418.

Heterosexual participants (33.5%) had significantly higher rates of sexual dysfunction than non-heterosexual participants (17.8%), X2(1)=9.470, p=.002, φ=-.174 (small effect size). Non-heterosexual participants had significantly higher total scores (M = 48.05, SD = 7.76) than heterosexual participants (M = 45.08, SD = 10.03), t(308.563)=-2.963, p=.003, d=-.325 (small effect size). In particular, non-heterosexual participants had significantly higher scores on the Desire/Frequency [t(331)=-3.278, p=.001, d=-.365] and Desire/Interest [t(318.257)=-4.612, p<.001, d=-.500] subscales than heterosexual participants. There was no significant difference in rates of sexual dysfunction or total or subscale CSFQ-14 scores between gay or lesbian and bisexual or pansexual participants.

Relationship status was not significantly associated sexual dysfunction. Relationship status was significantly associated with the CSFQ-14 total score (relationship: M = 47.10, SD = 8.90; dating or hooking up: M = 48.30, SD = 7.77; single: M = 43.32, SD = 10.21), F(2)=5.657, p=.004, η_p_^2^ = .035 (small effect size). Specifically, relationship status was significantly associated with the Pleasure subscale, F(2)=53.434, p<.001, η_p_^2^ = .245. Those who were in a relationship had similar Pleasure subscale scores to those who were dating or hooking up, and both had higher scores than those who were single.

Participants who were currently sexually active (had sexual experiences with at least one other person in the past month) were significantly less likely to meet criteria for sexual dysfunction (22.4%) than those who were not currently sexually active (37.4%), X^2^(1)=7.641, p=.006, φ=-.156 (small effect size). Participants who were currently sexually active had significantly higher CSFQ-14 total scores (M = 47.61, SD = 8.68) than those who were not (M = 43.43, SD = 9.93), F(1)=14.296, p<.001, η_p_^2^ = .044 (small effect size). In particular, those who were currently sexually active had significantly higher scores on the Pleasure [F(1)=182.309, p<.001, η_p_^2^ = .356] and Orgasm/Completion [F(1)=8.395, p=.004, η_p_^2^ = .026] subscales than those who were not.

Educational achievement was not associated with rates of sexual dysfunction. However, educational achievement was significantly associated with the CSFQ-14 total score (less than bachelor’s: M = 44.82, SD = 9.47; bachelor’s: M = 48.20, SD = 8.20; master’s or higher: M = 44.84, SD = 10.23), F(2)=5.274, p=.006, η_p_^2^ = .033 (small effect size). Educational achievement was also significantly associated with the Desire/Interest [F(2)=5.875, p=.003, η_p_^2^ = .034], Arousal/Excitement [F(2)=6.223, p=.002, η_p_^2^ = .037], and Orgasm/Completion [F(2)=6.109, p=.002, η_p_^2^ = .037] subscales. Participants with a bachelor’s degree had higher scores on all three of these subscales than those with less than bachelor’s degree and those with a master’s degree or higher.

Hispanic/Latino participants had significantly higher scores on the Desire/Frequency subscale than non-Hispanic/Latino participants, t(331)=-2.594, p=.010, d=-.458. Race and employment status were not significantly associated with rates of sexual dysfunction or CSFQ-14 total or subscales scores (**Table 1**).

**Table 1:** Demographics for 334 participants with trichotillomania, skin picking disorder, or both.

Demographic variable		Full sample	Sexual dysfunction		CSFQ-14 scores					
		All *M (SD)* or *%*	Dysfunctional *M (SD)* or *%*	Functional *M (SD)* or *%*	Total *M (SD)*	Pleasure *M (SD)*	Desire/Frequency *M (SD)*	Desire/Interest *M (SD)*	Arousal/Excitement *M (SD)*	Orgasm/Completion *M (SD)*
All (*n* = 334)		n/a	27.1%	72.9%	46.30 (9.27)	2.92 (1.23)	6.23 (1.69)	8.71 (2.64)	9.24 (2.72)	10.51 (3.41)
Age (*n* = 334)		30.21 (8.23)	31.72 (10.75)	30.43 (7.77)	n/a	n/a	n/a	n/a	n/a	n/a
Sex and gender (*n* = 334)	Cisgender women	83.5%	28.6%	71.4%	45.65 (9.48)	2.97 (1.24)	6.11 (1.69)	8.45 (2.59)	8.94 (2.62)	10.41 (3.59)
	Cisgender men	9.0%	14.3%	85.7%	53.32 (5.82)	2.97 (1.21)	7.13 (1.59)	10.80 (2.17)	12.21 (2.30)	11.41 (1.84)
	Gender minorities	7.5%	25.0%	75.0%	45.25 (6.65)	2.36 (1.08)	6.52 (1.42)	9.12 (2.65)	9.00 (2.21)	10.52 (2.62)
Ethnicity (*n* = 334)	Hispanic	10.8%	11.4%	48.94 (7.75)	11.4%	3.29 (1.32)	6.92 (1.59)	9.33 (2.16)	10.25 (2.70)	10.91 (3.05)
	Non-Hispanic	89.2%	29.0%	71.0%	45.97 (9.40)	2.88 (1.22)	6.15 (1.68)	8.64 (2.68)	9.11 (2.70)	10.46 (3.45)
Race (*n* = 330)	White	78.5%	25.3%	74.7%	46.39 (9.25)	2.95 (1.21)	6.14 (1.64)	8.68 (2.65)	9.25 (2.61)	10.63 (3.44)
	Black	5.5%	22.2%	77.8%	46.22 (7.77)	2.89 (1.37)	6.72 (1.87)	9.50 (2.75)	9.72 (2.80)	10.17 (2.48)
	Asian	10.9%	47.1%	52.9%	43.68 (10.33)	2.47 (1.21)	6.33 (2.03)	8.06 (2.75)	8.31 (3.10)	9.40 (3.66)
	MENA	1.8%	16.7%	83.3%	51.33 (8.12)	3.00 (1.26)	6.33 (.82)	10.33 (1.51)	11.67 (2.80)	11.67 (2.34)
	Other	3.3%	9.1%	90.9%	50.82 (7.49)	3.55 (1.44)	7.18 (1.60)	9.45 (1.51)	10.09 (2.84)	12.00 (3.03)
Sexual orientation (*n* = 334)	Heterosexual	59.0%	33.5%	66.5%	45.08 (10.03)	2.98 (1.27)	5.98 (1.73)	8.18 (2.73)	8.97 (2.95)	10.18 (3.67)
	Non-heterosexual	41.0%	17.8%	82.2%	48.05 (7.76)	2.84 (1.17)	6.59 (1.57)	9.47 (2.32)	9.62 (2.31)	10.98 (2.94)
Employment (*n* = 334)	Full-time	59.0%	26.6%	73.4%	46.30 (9.57)	2.97 (1.23)	6.11 (1.61)	8.50 (2.74)	9.09 (2.73)	10.64 (3.45)
	Part-time	9.3%	20.7%	79.3%	48.55 (7.62)	2.97 (1.11)	6.94 (1.86)	9.74 (2.22)	10.37 (2.39)	10.60 (3.22)
	Student	18.9%	33.3%	66.7%	44.67 (8.97)	2.60 (1.25)	6.24 (1.64)	8.69 (2.43)	8.92 (2.90)	9.87 (3.50)
	Homemaker	2.1%	14.3%	85.7%	46.29 (4.99)	3.71 (.95)	6.57 (.79)	8.43 (.98)	9.29 (1.50)	10.29 (1.60)
	Disabled	4.2%	35.7%	64.3%	45.71 (11.33)	3.29 (1.20)	5.79 (2.22)	8.64 (2.65)	9.07 (2.62)	11.07 (4.25)
	Unemployed	5.4%	18.8%	81.3%	48.81 (9.63)	2.89 (1.18)	6.39 (1.97)	9.17 (2.68)	10.00 (2.99)	11.12 (2.98)
	Other	1.2%	25.0%	75.0%	46.25 (6.65)	2.50 (1.91)	7.25 (1.71)	10.25 (4.27)	10.00 (1.63)	9.00 (2.16)
Education (*n* = 334)	Less than bachelor’s	31.4%	32.7%	67.3%	44.82 (9.47)	2.76 (1.29)	6.23 (1.88)	8.81 (2.64)	9.04 (2.80)	9.56 (3.77)
	Bachelor’s	42.5%	19.7%	80.3%	48.20 (8.20)	3.10 (1.18)	6.44 (1.54)	9.13 (2.45)	9.80 (2.57)	11.07 (2.87)
	Master’s or higher	26.0%	32.9%	67.1%	44.84 (10.23)	2.83 (1.22)	5.91 (1.64)	7.92 (2.78)	8.54 (2.71)	10.71 (3.57)
Relationship status (*n* = 334)	Relationship	64.7%	23.5%	76.5%	47.10 (8.90)	3.29 (1.14)	6.32 (1.56)	8.66 (2.63)	9.32 (2.62)	10.81 (3.21)
	Dating or hooking up	10.2%	24.2%	75.8%	48.30 (7.77)	3.18 (.97)	6.56 (1.56)	9.50 (2.30)	9.62 (2.69)	11.00 (2.33)
	Single	25.1%	37.7%	62.3%	43.32 (10.21)	1.87 (.92)	5.89 (1.99)	8.53 (2.76)	8.85 (2.99)	9.53 (4.07)
Current sexual activity* (*n* = 333)	Sexually active	67.6%	22.4%	77.6%	47.61 (8.68)	3.43 (1.03)	6.39 (1.48)	8.75 (2.58)	9.48 (2.58)	10.88 (3.06)
	Not sexually active	32.4%	37.4%	62.6%	43.43 (9.93)	1.86 (.91)	5.90 (2.02)	8.62 (2.77)	8.73 (2.97)	9.71 (3.98)

Ns may vary depending on whether there was missing data from participants not answering certain items. *Current sexual activity is defined as having sexual experiences with at least one other person in the past month. Sexual experiences is defined as any kind of sexual acts with other people, including kissing, touching, intercourse, or other sexual acts.

### 3.2 Pulling and picking details

There were no significant differences in pulling or picking variables between those who met criteria for sexual dysfunction and those who did not. The total CSFQ-14 score was also not significantly related to any pulling and picking variables.

Functional interference from pulling when pulling was at its worst was weakly negatively associated with the Pleasure subscale, r=-.224, p=.007, n=144. However, with controlling for relationship status, this result was no longer significant. After controlling for relationship status, participants who rated their picking as currently at its worst had significantly lower scores on the Orgasm/Completion subscale (M = 9.68) than those who said their picking had been worse before (M = 10.87), F(1)=6.838, p=.010, η_p_^2^ = .029 (small effect size) (**Table 2**).

**Table 2:** Pulling and picking details for 334 participants with trichotillomania, skin picking disorder, or both.

Pulling or picking variable			Full sample	Sexual dysfunction		CSFQ-14 scores					
			All *M (SD)* or *%*	Dysfunctional *M (SD)* or *%*	Functional *M (SD)* or *%*	Total *M (SD)*	Pleasure *M (SD)*	Desire/Frequency *M (SD)*	Desire/Interest *M (SD)*	Arousal/Excitement *M (SD)*	Orgasm/Completion *M (SD)*
Diagnosis (*n* = 334)	TTM only		27.2%	22.5%	77.5%	47.43 (9.38)	3.11 (1.20)	6.47 (1.73)	8.64 (2.61)	9.63 (2.85)	10.83 (3.35)
	SPD only		56.0%	25.9%	74.1%	46.51 (9.30)	2.81 (1.26)	6.20 (1.68)	8.74 (2.66)	9.16 (2.68)	10.64 (3.29)
	TTM and SPD		16.8%	38.2%	61.8%	43.82 (8.67)	2.98 (1.18)	5.96 (1.61)	8.75 (2.65)	8.86 (2.61)	9.56 (3.77)
Age on onset	TTM (*n* = 145)		13.76(5.22)	12.88(5.02)	14.08 (5.33)	n/a	n/a	n/a	n/a	n/a	n/a
	SPD (*n* = 237)		10.99 (7.09)	12.77 (9.79)	10.44 (5.76)	n/a	n/a	n/a	n/a	n/a	n/a
Duration of illness (years)	TTM (*n* = 145)		15.74 (9.10)	17.03 (8.48)	15.33 (9.39)	n/a	n/a	n/a	n/a	n/a	n/a
	SPD (*n* = 237)		19.47 (9.91)	19.64 (13.27)	19.07 (8.26)	n/a	n/a	n/a	n/a	n/a	n/a
Pulling in the past week (*n* = 147)	Days per week		5.80 (1.49)	5.90 (1.37)	5.79 (1.53)	n/a	n/a	n/a	n/a	n/a	n/a
	Time per day (minutes)		82.63 (50.00)	91.95 (96.36)	79.15 (88.45)	n/a	n/a	n/a	n/a	n/a	n/a
	How much pulling bothered (0-5)		3.69 (4.00)	3.90 (1.26)	3.62 (1.21)	n/a	n/a	n/a	n/a	n/a	n/a
	How much pulling interfered (0-5)		2.90 (1.36)	3.10 (1.34)	2.80 (1.37)	n/a	n/a	n/a	n/a	n/a	n/a
Picking in the past week (*n* = 243)	Days per week		6.31 (1.25)	6.26 (1.29)	6.32 (1.25)	n/a	n/a	n/a	n/a	n/a	n/a
	Time per day (minutes)		83.31 (111.73)	76.46 (87.06)	82.00 (113.36)	n/a	n/a	n/a	n/a	n/a	n/a
	How much picking bothered (0-5)		3.87 (1.15)	3.98 (1.22)	3.86 (1.10)	n/a	n/a	n/a	n/a	n/a	n/a
	How much picking interfered (0-5)		2.99 (1.30)	3.09 (1.28)	2.94 (1.30)	n/a	n/a	n/a	n/a	n/a	n/a
Pulling at its worst	Currently at worst (*n* = 144)	Yes	50.7%	23.9%	76.1%	46.68 (8.45)	3.10 (1.11)	6.21 (1.70)	8.62 (2.46)	9.36 (2.77)	10.99 (3.22)
		No	49.3%	32.4%	67.6%	45.48 (10.10)	3.07 (1.27)	6.37 (1.73)	8.70 (2.82)	9.27 (2.80)	9.79 (3.81)
	Days per week (*n* = 144)		6.48 (1.07)	6.53 (1.01)	6.47 (1.10)	n/a	n/a	n/a	n/a	n/a	n/a
	Time per day (minutes) (*n* = 142)		112.14 (122.14)	136.67 (163.77)	102.91 (102.31)	n/a	n/a	n/a	n/a	n/a	n/a
	How much pulling bothered (0-5) (*n* = 144)		4.28 (1.02)	4.43 (1.08)	4.25 (.99)	n/a	n/a	n/a	n/a	n/a	n/a
	How much pulling interfered (0-5) (*n* = 144)		3.63 (1.20)	4.00 (1.01)	3.47 (1.25)	n/a	n/a	n/a	n/a	n/a	n/a
Picking at worst	Currently at worst (*n* = 237)	Yes	40.5%	34.8%	65.2%	44.42 (10.14)	2.74 (1.23)	6.01 (1.72)	8.64 (2.75)	8.95 (2.98)	9.68 (3.80)
		No	59.5%	24.8%	75.2%	46.71 (8.39)	2.91 (1.26)	6.21 (1.65)	8.71 (2.57)	9.12 (2.43)	10.87 (3.10)
	Days per week (*n* = 237)		6.68 (.88)	6.55 (1.05)	6.72 (.82)	n/a	n/a	n/a	n/a	n/a	n/a
	Time per day (minutes) (*n* = 236)		120.00 (144.27)	106.80 (113.55)	116.66 (135.29)	n/a	n/a	n/a	n/a	n/a	n/a
	How much picking bothered (0-5) (*n* = 236)		4.37 (.97)	4.34 (1.09)	4.41 (.85)	n/a	n/a	n/a	n/a	n/a	n/a
	How much picking interfered (0-5) (*n* = 234)		3.75 (1.25)	3.81 (1.22)	3.72 (1.28)	n/a	n/a	n/a	n/a	n/a	n/a

Ns may vary depending on whether participants met criteria for TTM, SPD, or both and whether there was missing data from participants not answering certain items.

Age of onset and duration of illness was not significantly associated with sexual dysfunction, the CSFQ-14 total score, or any subscales.

### 3.3 Current comorbidities and treatments

Of the entire sample, 75.2% endorsed currently experiencing symptoms of a comorbid disorder. The most common comorbidities were anxiety disorders (58.9%), depression (38.5%), ADHD (36.3%), OCD (17.8%), and PTSD (11.5%). Sexual dysfunction, the total CSFQ-14 score, and subscale scores were not significantly associated with any current comorbidities (**Table 3**).

**Table 3:** Current comorbidities for 334 participants with trichotillomania, skin picking disorder, or both.

Comorbidity		Full sample	Sexual dysfunction		CSFQ-14 scores					
		All *%*	Dysfunctional *%*	Functional *%*	Total *M (SD)*	Pleasure *M (SD)*	Desire/Frequency *M (SD)*	Desire/Interest *M (SD)*	Arousal/Excitement *M (SD)*	Orgasm/Completion *M (SD)*
Any (*n* = 314)	Yes	75.2%	26.9%	73.1%	45.87 (8.93)	2.89 (1.25)	6.21 (1.65)	8.59 (2.58)	9.12 (2.58)	10.50 (3.45)
	No	24.8%	25.7%	74.3%	47.50 (10.01)	3.01 (1.28)	6.25 (1.83)	9.59 (3.09)	9.59 (3.09)	10.59 (3.41)
Anxiety (*n* = 314)	Yes	58.9%	26.4%	73.6%	46.02 (9.07)	2.82 (1.26)	6.26 (1.61)	8.58 (2.64)	9.13 (2.65)	10.57 (3.48)
	No	41.1%	26.8%	73.2%	46.63 (9.46)	3.05 (1.24)	6.16 (1.81)	8.80 (2.64)	9.38 (2.82)	10.44 (3.38)
Depression (*n* = 314)	Yes	38.5%	31.3%	68.7%	45.64 (9.68)	2.85 (1.24)	6.30 (1.71)	8.83 (2.57)	9.03 (2.79)	10.22 (3.63)
	No	61.5%	23.6%	76.4%	46.68 (8.93)	2.96 (1.27)	6.17 (1.69)	8.57 (2.68)	9.36 (2.67)	10.71 (3.31)
ADHD (*n* = 314)	Yes	36.3%	23.4%	76.6%	46.78 (8.19)	2.96 (1.28)	6.39 (1.54)	8.75 (2.69)	9.20 (2.42)	10.60 (3.17)
	No	63.7%	28.4%	71.6%	45.99 (9.77)	2.89 (1.24)	6.12 (1.77)	8.63 (2.61)	9.26 (2.88)	10.47 (3.59)
OCD (*n* = 314)	Yes	17.8%	22.2%	77.8%	45.28 (9.68)	2.79 (1.11)	6.11 (1.89)	8.98 (2.60)	9.00 (2.70)	10.00 (3.53)
	No	82.2%	27.6%	72.4%	46.50 (9.13)	2.95 (1.29)	6.24 (1.65)	8.60 (2.65)	9.29 (2.73)	10.63 (3.41)
PTSD (*n* = 314)	Yes	11.5%	26.5%	73.5%	44.32 (10.29)	2.58 (1.30)	5.83 (1.92)	8.44 (2.61)	8.63 (2.94)	9.89 (3.91)
	No	88.5%	26.6%	73.4%	46.53 (9.07)	2.96 (1.25)	6.27 (1.66)	8.70 (2.64)	9.31 (2.69)	10.60 (3.37)
Eating disorder (*n* = 314)	Yes	5.4%	23.5%	76.5%	46.12 (8.57)	2.76 (1.39)	6.65 (1.27)	9.24 (3.35)	9.47 (2.43)	10.35 (2.96)
	No	94.6%	26.8%	73.2%	46.29 (9.28)	2.93 (1.25)	6.19 (1.71)	8.64 (2.59)	9.22 (2.74)	10.53 (3.47)
Personality disorder (*n* = 314)	Yes	3.8%	27.3%	72.7%	45.73 (6.45)	2.67 (1.37)	6.42 (1.44)	9.00 (2.80)	9.91 (2.02)	9.08 (3.20)
	No	96.2%	26.6%	73.4%	46.30 (9.32)	2.93 (1.25)	6.21 (1.70)	8.66 (2.63)	9.21 (2.74)	10.58 (3.44)
Bipolar (*n* = 314)	Yes	2.5%	50.0%	50.0%	42.50 (6.50)	2.25 (1.16)	5.50 (1.60)	8.00 (2.33)	8.13 (1.36)	10.13 (3.00)
	No	97.5%	26.0%	74.0%	46.38 (9.27)	2.93 (1.25)	6.24 (1.69)	8.69 (2.65)	9.26 (2.74)	10.53 (3.45)
AUD (*n* = 314)	Yes	1.0%	0%	100%	50.33 (7.64)	3.00 (1.00)	7.67 (1.53)	11.33 (2.89)	10.67 (2.08)	10.67 (2.52)
	No	99.0%	26.9%	73.1%	46.23 (7.64)	2.92 (1.26)	6.20 (1.69)	8.65 (2.63)	9.22 (2.72)	10.52 (3.45)
SUD (*n* = 314)	Yes	0.6%	50.0%	50.0%	47.00 (15.56)	1.50 (.71)	6.50 (3.54)	8.50 (4.95)	10.00 (5.66)	11.50 (2.12)
	No	99.4%	26.4%	73.6%	46.27 (9.21)	2.93 (1.25)	6.22 (1.69)	8.67 (2.63)	9.23 (2.71)	10.51 (3.44)
Schizophrenia (*n* = 314)	Yes	0%	n/a	n/a	n/a	n/a	n/a	n/a	n/a	n/a

Ns may vary depending on whether there was missing data from participants not answering certain items.

69.5% of the full sample indicated that they were receiving current psychiatric treatment, with 56.3% currently receiving medication and 42.2% currently in therapy. The mean number of current psychiatric mediations taken was 1.07 (SD = 1.23). 45.8% of the sample were currently taking antidepressants, 22.3% taking stimulants, 8.7% taking anxiolytics, 5.8% taking antipsychotics, 5.8% taking anticonvulsants, and 7.1% taking another type of psychiatric medication. 36.2% were currently taking hormonal medications. None of these treatment variables were significantly associated with sexual dysfunction, the total CSFQ-14 score, or any subscale scores (**Table 4**).

**Table 4:** Current treatments for 334 participants with trichotillomania, skin picking disorder, or both.

Treatment variable			Full sample	Sexual dysfunction		CSFQ-14 scores					
			All *M (SD)* or *%*	Dysfunctional *M (SD)* or *%*	Functional *M (SD)* or *%*	Total *M (SD)*	Pleasure *M (SD)*	Desire/Frequency *M (SD)*	Desire/Interest *M (SD)*	Arousal/Excitement *M (SD)*	Orgasm/Completion *M (SD)*
Current psychiatric treatment	Any (*n* = 302)	Yes	69.5%	26.5%	73.5%	46.19 (9.15)	2.98 (1.28)	6.23 (1.60)	8.56 (2.60)	9.15 (2.60)	10.60 (3.47)
		No	30.5%	29.2%	70.8%	46.09 (9.50)	2.72 (1.23)	6.13 (1.93)	8.68 (2.66)	9.37 (2.97)	10.36 (3.43)
	Medication (*n* = 300)	Yes	56.3%	28.3%	71.7%	45.82 (9.56)	2.90 (1.30)	6.14 (1.62)	8.49 (2.68)	9.02 (2.65)	10.55 (3.54)
		No	43.7%	26.6%	73.4%	46.51 (8.90)	2.87 (1.22)	6.24 (1.80)	8.69 (2.52)	9.44 (2.77)	10.48 (3.38)
	Therapy (*n* = 301)	Yes	42.2%	25.0%	75.0%	46.34 (9.27)	3.01 (1.30)	6.16 (1.62)	8.46 (2.56)	9.16 (2.69)	10.77 (3.43)
		No	57.8%	29.2%	70.8%	45.93 (9.18)	2.81 (1.24)	6.22 (1.77)	8.68 (2.64)	9.23 (2.71)	10.33 (3.47)
Current psychiatric medications	Number of current medications (*n* = 297)		1.08 (1.22)	1.14 (1.22)	1.07 (1.24)	n/a	n/a	n/a	n/a	n/a	n/a
	Antidepressant (*n* = 298)	Yes	46.0%	31.3%	68.8%	45.16 (9.93)	2.85 (1.31)	6.05 (1.61)	8.49 (2.80)	8.95 (2.71)	10.23 (3.70)
		No	54.0%	24.8%	75.2%	46.86 (8.67)	2.91 (1.23)	6.30 (1.79)	8.63 (2.45)	9.43 (2.71)	10.71 (3.24)
	Stimulant (*n* = 298)	Yes	22.5%	22.6%	77.4%	47.03 (8.35)	2.85 (1.40)	6.52 (1.69)	8.66 (2.54)	9.31 (2.38)	10.80 (2.93)
		No	77.5%	29.2%	70.8%	45.82 (9.54)	2.89 (1.23)	6.09 (1.70)	8.54 (2.63)	9.18 (2.81)	10.41 (3.60)
	Anxiolytic (*n* = 298)	Yes	9.1%	22.6%	77.4%	46.48 (10.58)	3.07 (1.36)	6.44 (1.85)	8.85 (2.52)	9.56 (2.79)	10.04 (4.01)
		No	90.9%	29.2%	70.8%	46.04 (9.16)	2.86 (1.26)	6.16 (1.69)	8.54 (2.62)	9.17 (2.71)	10.54 (3.41)
	Antipsychotic (*n* = 298)	Yes	5.7%	35.3%	64.7%	43.88 (11.71)	2.53 (1.50)	6.00 (2.03)	8.24 (2.86)	8.65 (3.26)	9.65 (4.21)
		No	94.3%	27.3%	72.7%	46.23 (9.12)	2.90 (1.25)	6.20 (1.69)	8.59 (2.60)	9.24 (2.68)	10.55 (3.41)
	Anticonvulsant (*n* = 298)	Yes	6.0%	27.8%	72.7%	45.00 (12.43)	2.61 (1.42)	6.33 (2.33)	8.83 (3.19)	9.00 (3.12)	9.83 (4.19)
		No	94.0%	27.8%	72.2%	46.16 (9.06)	2.90 (1.25)	6.18 (1.67)	8.55 (2.57)	9.22 (2.69)	10.54 (3.41)
	Other (*n* = 298)	Yes	7.0%	28.6%	71.4%	47.29 (8.50)	2.81 (1.25)	6.71 (1.35)	8.33 (2.74)	9.67 (2.82)	10.95 (3.11)
		No	93.0%	27.7%	72.3%	45.99 (9.36)	2.89 (1.27)	6.15 (1.73)	8.58 (2.60)	9.17 (2.71)	10.46 (3.49)
Current hormonal medication use (*n* = 301)		Yes	36.2%	28.4%	71.6%	45.70 (10.20)	2.97 (1.26)	6.25 (1.72)	8.26 (2.77)	9.06 (2.77)	10.38 (3.53)
		No	63.8%	26.9%	73.1%	46.41 (8.71)	2.85 (1.27)	6.16 (1.70)	8.79 (2.52)	9.32 (2.69)	10.60 (3.43)

Ns may vary depending on whether there was missing data from participants not answering certain items.

## 4 Discussion

In this largely female sample, approximately 1 in 4 adults with trichotillomania, skin picking disorder, or both met criteria for sexual dysfunction. These results should be considered cautiously given that this study’s sample was majority non-Hispanic white and had high educational attainment. In particular, greater educational achievement has been found to be associated with greater sexual functioning in previous studies as well as in this sample. Similarly, while the proportion of partnered subjects in this sample is in line with estimates of relationship status in the general U.S. population, those with college degrees are more likely to be in relationships (30). Given that those in relationships tend to have better sexual functioning, the prevalence of sexual dysfunction in a more representative sample of those with trichotillomania and skin picking disorder may be higher. Therefore, the findings from this study should not be taken as epidemiological estimates, but rather as starting points for future research that can explore the hypotheses discussed here.

This study also demonstrates that sexual functioning is correlated with certain measures of severity of skin picking. Participants who indicated that their skin picking was currently at its worst had worse orgasmic functioning. Thus, participants with more severe cases of skin picking disorder may also have more issues with sexual functioning. Further research is necessary to corroborate this study's findings that there was no link between trichotillomania severity and sexual functioning.

Prior research indicates that orgasmic dysfunction is the most common sexual problem in adults with OCD, including being unable to achieve an orgasm and reduced quality of orgasm (9, 11, 12, 31, 32). In this study, the orgasm domain of sexual functioning also appears to be the most closely linked to skin picking disorder symptoms, suggesting that there may be shared features across obsessive-compulsive and related disorders that impact sexual functioning in similar ways. However, there were also several ways in which the results in this study diverge from prior research on the relationship between sexual functioning and psychiatric disorders. There was no relationship in this study between duration of illness and sexual functioning, nor between pharmacological or psychotherapeutic treatment and sexual functioning. Most interestingly, neither sexual dysfunction nor the level of sexual functioning were associated with any current psychiatric comorbidities in this sample, suggesting that comorbid trichotillomania or skin picking disorder may complicate the relationship between other psychiatric disorders and sexual functioning.

There are several limitations to this study that should be considered in interpreting these results. Primarily, this is a self-report study where participants self-identified as having trichotillomania or skin picking disorder, and we did not use a validated scale of hair pulling or skin picking severity (although the items we used such as time spent picking/pulling and interference are standard ways of assessing severity). This method introduces a self-selection bias; therefore, the results of this study may not be generalizable to clinical or population-based samples. Additionally, there was no examination of the temporal relationship between starting pulling/picking and sexual dysfunction, so causality could not be determined. Participants also self-reported other psychiatric disorders, and we only used dichotomous variables to stratify participants into those who did and did not have a diagnosis, rather than using a validated diagnostic or severity measure. The finding that comorbidities were not associated with sexual dysfunction should be interpreted with caution, as those with more severe comorbidities in the sample may be more likely to present with sexual dysfunction. Similarly, we examined current medication use dichotomously in this sample; however, we did not examine the duration the medication was taken, the dose, or other related variables.

There were many variables that have been found to predict sexual functioning in prior research that we did not collect data on in this study, such as health issues, relationship satisfaction, and sexual history. There were also factors specific to trichotillomania and skin picking disorder that we did not examine in this study. For example, pulling or picking from the genital area may be associated with more sexual dysfunction than pulling or picking from other areas. Furthermore, while we generally asked participants about distress and functional interference, asking more specific questions about how pulling and picking have impacted appearance-related concerns may be helpful. Future research on sexual functioning in trichotillomania and skin picking disorder should study these variables. Finally, sociodemographic factors have a large impact on the types and quality of sexual experiences, and the prevalence of sexual dysfunction can vary greatly between countries as well as between different groups in the same country. In this study, several demographic factors were correlated with sexual functioning, including sex and gender, sexual orientation, ethnicity, and education. Given that this sample was from the U.S., majority female, majority non-Hispanic white, and highly educated, we may not be able to generalize these findings to other demographic groups.

In conclusion, it is difficult to compare the rate of sexual dysfunction in adults with trichotillomania and skin picking disorder with the rate in the general population given the homogeneity of this study’s sample. Moreover, the findings from this study should be viewed as preliminary given that we did not use a validated scale to measure pulling and picking severity. Future research is also needed to determine whether the severity of comorbid diagnoses have an effect on sexual functioning in this population. Having said that, the rates of sexual dysfunction in this study suggest a significant and overlooked issues among adults with trichotillomania and skin picking disorder. Further research is required to determine what can be done to improve sexual functioning in people with these disorders.

## Data Availability

The original contributions presented in the study are included in the article/supplementary material. Further inquiries can be directed to the corresponding author.
